# Vitamin D and Immune Checkpoint Inhibitors in Lung Cancer: A Synergistic Approach to Enhancing Treatment Efficacy

**DOI:** 10.3390/ijms26104511

**Published:** 2025-05-09

**Authors:** Yu Zhang, Yan Xu, Wei Zhong, Jing Zhao, Xiaoyan Liu, Xiaoxing Gao, Minjiang Chen, Mengzhao Wang

**Affiliations:** Department of Respiratory & Critical Care Medicine, Peking Union Medical College Hospital, Chinese Academy of Medical Sciences, Peking Union Medical College, Beijing 100730, China; zoe_yuzhang0513@163.com (Y.Z.); maraxu@163.com (Y.X.); zw_pumch@126.com (W.Z.); pumchzj@sina.com (J.Z.); liuxiaoyan@pumch.cn (X.L.); claregao@126.com (X.G.); mengzhaowang@sina.com (M.W.)

**Keywords:** vitamin D, immune checkpoint inhibitors, synergistic effect, lung cancer

## Abstract

Lung cancer, a malignant neoplasm that is globally prevalent and characterized by high incidence and mortality rates, has seen the rise of immune checkpoint inhibitors (ICIs) as a crucial systemic treatment. However, a subset of patients exhibits suboptimal responses to ICIs. Recently, studies revealed the role of vitamin D in inflammation modulation, cell differentiation, and cancer prevention. Vitamin D precisely modulates immune responses and inflammatory states within the tumor microenvironment (TME) by targeting both innate and adaptive immunity. These effects may reduce immune tolerance to ICIs and synergistically enhance their therapeutic efficacy. Here, we review vitamin D metabolism in lung cancer patients, as well as its anti-tumor mechanisms, immune regulation, and the significant promise of vitamin D in lung cancer immunotherapy and adjuvant therapeutic strategies. Further research is imperative to surmount these challenges and fully realize vitamin D’s potential in improving lung cancer immunotherapy outcomes.

## 1. Introduction

The incidence and mortality rates of cancer remain a significant global health challenge, with lung cancer being one of the most prevalent and deadly malignancies globally. GLOBOCAN 2020 estimates reveal that nearly 1.81 million individuals succumbed to lung cancer, representing approximately 18% of all cancer-related fatalities globally [1,2]. Over the past few years, immunotherapy has emerged as a groundbreaking approach in lung cancer treatment, especially using immune checkpoint inhibitors (ICIs). These therapies have demonstrated remarkable efficacy in extending survival rates and improving patients’ quality of life [3,4,5]. Despite these advances, a substantial proportion of lung cancer patients fail to respond adequately to immunotherapy, highlighting the urgent need for innovative strategies [6,7].

Vitamin D is renowned for its classical role in calcium homeostasis. Emerging evidence also suggested that vitamin D also possesses anticancer properties, influencing tumor initiation, progression, and metastasis [8,9,10]. Beyond these functions, vitamin D exhibits significant immunomodulatory effects. It regulates the differentiation and function of immune cells and modulates inflammatory responses [11]. Moreover, vitamin D has been shown to have the ability to influence the tumor microenvironment (TME) by reducing immunosuppressive cell populations, including myeloid-derived suppressor cells (MDSCs), while enhancing the activity of cytotoxic T cells [12,13,14]. Several studies have clearly indicated that vitamin D might potentiate the antitumor effectiveness of ICIs [15,16]. A prospective controlled study indicated that patients with cutaneous malignancies who possessed adequate vitamin D levels exhibited markedly improved responses to anti-programmed death-1 (PD-1) immunotherapy in comparison to those with vitamin D insufficiency [15]. Similarly, the PROVIDENCE study discovered that the survival and disease control rates of patients with advanced solid tumors who were receiving ICIs were enhanced by systemic vitamin D ingestion [16]. These data collectively indicate that vitamin D may serve as a valuable adjunct to immunotherapy, and its potential role in lung cancer immunotherapy necessitates further investigation. This review thoroughly clarifies the synergistic interactions of vitamin D and ICIs in lung cancer therapy and offers insights into the utilization of vitamin D as a synergistic agent in cancer immunotherapy.

## 2. Immune Checkpoint Inhibitors (ICIs)

**Figure 1:**
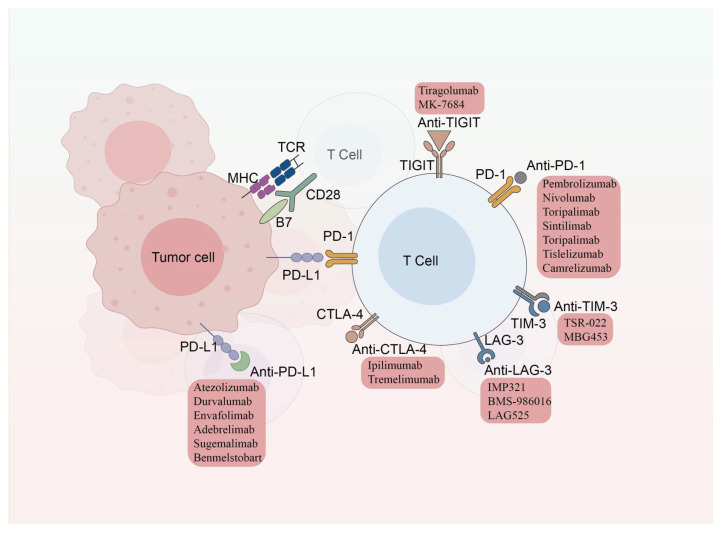
Categories of immune checkpoint inhibitors.

In recent years, ICIs have revolutionized lung cancer treatment, with three main classes currently approved: PD-1 inhibitors (nivolumab, pembrolizumab, sintilimab), programmed death-ligand 1 (PD-L1) inhibitors (atezolizumab, durvalumab), and cytotoxic T-lymphocyte-associated protein-4 (CTLA-4) inhibitors (ipilimumab, tremelimumab) [17]. Clinical trials such as KEYNOTE-024/042 and KEYNOTE-189/407 have established immunotherapy alone or combined with chemotherapy as the standard first-line regimen in non-small cell lung cancer (NSCLC) [18,19]. In small cell lung cancer (SCLC), the Impower 133 study and CASPIAN study have established chemoimmunotherapy as the standard first-line regimen [20,21]. Recent advances in both monotherapy and combination strategies (e.g., dual checkpoint blockade) continue to improve clinical outcomes. Emerging bispecific antibodies (e.g., cadonilimab) and novel agents targeting T cell immunoreceptor with immunoglobulin and ITIM domains (TIGIT) (e.g., Tiragolumab, MK-7684), lymphocyte activation gene-3(LAG-3) (e.g., IMP321, BMS-986016, LAG525), and T cell immunoglobulin and mucin domain containing-3 (TIM-3) (e.g., TSR-022, MBG453) are under clinical evaluation to overcome resistance in advanced NSCLC [22]. These emerging inhibitors show great potential to significantly boost the treatment outcomes for advanced NSCLC. For additional details, please refer to Figure 1 and Supplementary Table S1.

## 3. Vitamin D

### 3.1. The Synthesis Process of Vitamin D

Vitamin D occurs in several isomeric forms. The two principal isomers are vitamin D_2_ and vitamin D_3_. Vitamin D_2_ is not capable of being synthesized by the human body and is primarily derived from plant and fungal sources. In contrast, vitamin D_3_ can be produced via several biosynthetic pathways. In humans, 7-dehydrocholesterol can be converted into vitamin D_3_ through ultraviolet irradiation. Additionally, animal-based foods serve as a crucial source of dietary vitamin D_3_. It is derived from food and is absorbed into the bloodstream via chylomicrons after undergoing emulsification in the small intestinal tract. Skin-synthesized vitamin D is initially stored in adipose tissue, from which it is gradually released into the bloodstream. Upon its initial release into circulation, vitamin D is not physiologically active. Subsequently, it undergoes a two-step hydroxylation process in the liver and kidney to be converted into its bioactive form. In the liver, 25-hydroxylases (CYP2R1 and CYP27A1) convert it to 25(OH)D, a marker of vitamin D levels [23]. Then, 25(OH)D further converts to 1,25(OH)_2_D_3_ through the action of the renal CYP27B1 enzyme [24]. Besides the above classical pathway, the CYP11A1-involved alternative pathway catalyzes sequential hydroxylation of vitamin D, generating over 20 hydroxylated metabolites that exhibit diverse and significant biological activities [25,26].

### 3.2. The Catabolic Process and Its Regulatory Mechanisms

The metabolism and control of vitamin D constitute a sophisticated process involving numerous essential enzymes, hormones, and feedback systems. These elements work in concert to maintain the dynamic equilibrium of vitamin D homeostasis. 24-hydroxylase (CYP24A1), 1α-hydroxylase, and parathyroid hormone (PTH) are crucial in this process. CYP24A1 serves as an enzyme in the catabolic pathway of vitamin D, facilitating the degradation of both 25(OH)D and 1,25(OH)_2_D_3_, thereby precisely regulating the concentrations of these two vitamin D metabolites within the body. These metabolites can alter their own metabolism through feedback mechanisms. Furthermore, 1,25(OH)_2_D_3_ adjusts PTH release via a negative feedback loop, maintaining vitamin D metabolic homeostasis [27,28].

### 3.3. Physiological Function

1,25(OH)_2_D_3_ performs essential biological functions predominantly through the calcitriol-vitamin D receptor (VDR) pathway, which is classically referred to as the calcitriol-VDR pathway [29]. Vitamin D metabolites, generated through CYP11A1, possess the capability to specifically bind to the VDR. This binding facilitates a range of molecular and biological processes [30]. VDR is of vital significance in regulating calcium and phosphorus metabolism, as well as in muscle cell development. It is important to highlight that VDR is not merely found in conventional target tissues. It is widely distributed in non-target tissues as well, including epithelial and immune cells. Besides VDR, RORα and RORγ, isoforms of the ROR subfamily, can bind to CYP11A1-derived vitamin D metabolites. This CYP11A1-derived vitamin D metabolite-RORα/γ pathway regulates immune and metabolic responses associated with diseases including cancer and autoimmunity [30,31].

## 4. The Interrelation Between Vitamin D and Lung Cancer

### 4.1. Impairment of Vitamin D Metabolism in Individuals with Lung Cancer

In individuals diagnosed with lung carcinoma, a prevalent disorder in vitamin D metabolism is observed [32,33,34]. A number of studies have solidly confirmed a significant connection between smoking and lung cancer [35,36]. Benzo[a]pyrene (BaP), a primary harmful compound generated during cigarette combustion, has been demonstrated to induce the expression of 24-hydroxylase [32]. However, when the expression level of 24-hydroxylase is upregulated, its catalytic activity increases, consequently accelerating the catabolic process of 1,25(OH)_2_D_3_ and ultimately resulting in the production of inactive 24,25-(OH)_2_D_3_. Shiratsuchi et al. demonstrated that CYP24A1 is frequently overexpressed in tumor tissues. This elevated expression facilitates the conversion of bioactive 1,25(OH)_2_D_3_ into its inactive metabolite, leading to a decline in circulating bioactive vitamin D concentrations [33]. Moreover, research on lung carcinoma patients indicated that serum 25-(OH) D levels progressively decline in parallel with tumor stage progression in pulmonary malignancy patients [34]. This metabolic abnormality implies that patients with lung cancer may obtain more significant benefits from therapeutic strategies incorporating vitamin D, emphasizing the promising real-world applications of vitamin D studies in those patients.

### 4.2. The Anti-Tumor Mechanism of Vitamin D

The mechanisms underlying the impacts of vitamin D on cancer cells are intricate and multifaceted. It precisely regulates tRNA-derived small RNAs (tsRNAs), inducing mitochondrial dysfunction in lung cancer cells, effectively inhibiting their proliferation, and accelerating the apoptotic process [37]. Furthermore, it exerts a significant influence on the matrix within the TME as well as on cancer stem cells [38]. Vitamin D exhibits dose-dependent downregulation of key determinants, including vascular endothelial growth factor (VEGF) and matrix metalloproteinases (MMPs). The regulatory system markedly inhibits angiogenesis and reduces matrix degradation, thereby lessening the migratory, invasive, and angiogenic capabilities of lung cancer cells [39]. By inactivating the PI3K/AKT/mTOR signaling route, vitamin D dismantles the critical signaling network that sustains the stemness of lung cancer cells, suppressing their differentiation potential and impeding their ability to maintain an undifferentiated, malignant proliferative state. Additionally, vitamin D significantly inhibits the Warburg effect, disrupting glycolytic metabolism in tumor cells and consequently depleting the energy supply essential for their growth and proliferation [40].

### 4.3. Vitamin D Status and Prognosis in Lung Carcinoma: Clinical Correlation Studies

Meta-analytic evidence has demonstrated a notable linkage between decreased plasma 25(OH)D levels and adverse clinical outcomes, including poor prognosis and reduced survival rates [41]. The administration of vitamin D supplements to maintain adequate circulating 25(OH)D levels might potentially lower the mortality risk in this patient population [42,43]. Moreover, various investigations have revealed that administering vitamin D supplements can boost the survival probabilities of patients in the early stage of lung carcinoma, specifically for individuals presenting with suboptimal 25(OH)D concentrations [44,45] (Table 1). Contrary to earlier reports, multivariate analysis of observational data revealed no significant correlation between circulating 25(OH)D levels and overall survival in individuals with metastatic pulmonary carcinoma [46,47]. This discrepancy may be attributed to the generally poor overall survival rates among patients with advanced lung carcinoma, which may minimize the influence of fluctuations in vitamin D levels on the overall progression of the disease. Therefore, ensuring sufficient 25(OH)D levels may be critically important for enhancing the prognosis of patients; however, its precise role may differ based on the disease stage and individual variability among patients.

### 4.4. The Influence of Vitamin D on the Efficacy and Adverse Effects of ICIs

A prospective controlled study demonstrated that patients with cutaneous malignancies who had sufficient vitamin D levels showed significantly better responses to ICIs than those with vitamin D deficiency [15]. The PROVIDENCE study found that for patients with advanced solid tumors undergoing ICI therapy, systemic intake of vitamin D could improve their survival rates and the control of the disease [16]. A prospective cohort study revealed that individuals with metastatic pulmonary carcinoma who received ICIs displayed significantly higher baseline levels of 25(OH)D among those achieving partial response (PR) compared to those who did not achieve PR [48]. These findings imply a potential connection between initial 25(OH)D status and the therapeutic outcome of ICIs, as well as patient prognosis. An accurate evaluation of baseline vitamin D levels in lung carcinoma patients, along with suitable supplementation based on these assessments, may serve as a useful technique to enhance the therapeutic efficacy of ICIs in clinical practice. Vitamin D can affect drug pharmacokinetics by modulating gene expression related to drug metabolism and elimination. Jessica Cusato’s investigation revealed a statistically significant correlation between hypovitaminosis D and altered nivolumab pharmacokinetics, suggesting that vitamin D repletion strategies may modulate the drug’s metabolism, distribution, and elimination to optimize therapeutic outcomes [49]. Professor Rahma’s team has demonstrated that vitamin D intake greatly reduces the occurrence of ICI-induced colitis in metastatic melanoma patients [50]. Similarly, a recent investigation demonstrated that elevated pretreatment 25(OH)D concentrations correlate with a reduced incidence of immune-related adverse events (irAEs) in advanced lung carcinoma patients using ICIs. This indicates that vitamin D can mitigate irAEs, reduce treatment interruptions, extend the duration of immunotherapy, and allow patients to continue benefiting from it [48].

The studies mentioned above (Table 2) delved into the impact of vitamin D on the efficacy and adverse effects of ICIs, with the aim of enhancing cancer treatment strategies. In summary, vitamin D exerts effects on modulating the efficacy and safety of immunotherapy for lung cancer via multi-faceted mechanisms, including immunomodulation, pharmacokinetic optimization, and toxicity regulation. Baseline vitamin D levels show potential as a biomarker for predicting the effectiveness of ICIs. In clinical practice, precise evaluation of baseline vitamin D status combined with personalized supplementation strategies may represent innovative approaches to optimize ICI therapy—enhancing antitumor efficacy, mitigating treatment-related toxicity, and extending the duration of immunotherapy benefits for patients.

## 5. The Mechanism Through Which Vitamin D Enhances the Effectiveness of ICIs in Lung Cancer Patients

**Figure 2:**
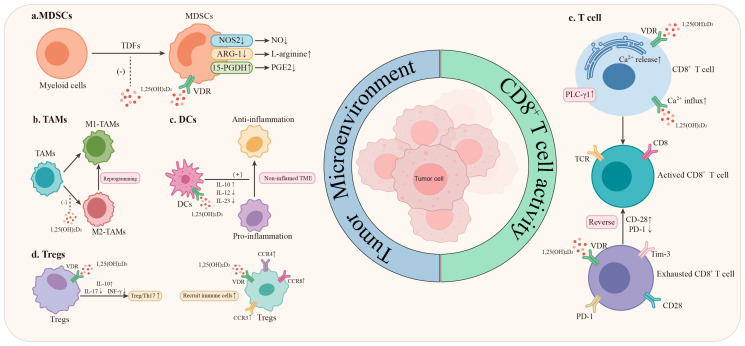
The potential mechanisms of vitamin D in synergizing with the efficacy of immune checkpoint inhibitors (ICIs). As presented in (**a**–**d**), vitamin D exerts its influence on immune cells, diminishing the immunosuppressive state within the tumor microenvironment, which subsequently elevates the therapeutic efficacy of ICIs; As illustrated in (**e**), vitamin D is capable of boosting the activity of CD8^+^ T cells, reinforcing the cytotoxic function of T cells and thus further augmenting the effectiveness of ICIs.

Tumor cells, through a variety of complex mechanisms such as maintaining continuous high expression of immune checkpoint molecules, successfully evade immune recognition and attack, achieving immune escape [51]. ICIs enhance the immune system’s capacity to eradicate tumor cells by obstructing molecular signaling pathways. However, the response rate is limited by the intricate and heterogeneous nature of the TME [7]. It is crucial to emphasize that a broad spectrum of immune cells, including T cells and macrophages, express both the VDR and 1α-hydroxylase. This dual-expression pattern confers these immune cells with highly significant functions. Firstly, they serve as targets for vitamin D, directly responding to its regulatory signals and playing a crucial role in the immune response. Secondly, these immune cells possess the capacity to locally activate vitamin D within tissues. Through 1α-hydroxylase, they metabolize vitamin D into a form with enhanced biological activity, thereby exerting more precise and efficient intracellular regulation. This further augments the performance of immune cells and supports the proper progression of the immune response [52]. Vitamin D within these immune cells exhibits a range of effects, such as modulating immune responses, suppressing excessive inflammatory reactions, and mitigating oxidative stress [8,37]. In relation to the field of lung cancer immunotherapy, this paper will systematically summarize how vitamin D modulates immune responses and inflammatory conditions within the TME via multiple targets of innate and adaptive immunity, ultimately enhancing the efficacy of ICIs (Figure 2).

### 5.1. Innate Immunity System

The innate immune response, acting as the body’s primary line of defense and initial barrier against invading pathogens and abnormal cells, is rapidly triggered in the tumor immune response. This immediate activation signifies the onset of the body’s anti-cancer defense mechanisms, initiating the earliest processes for combating tumors. These processes play a critical role in identifying and eradicating tumor cells before they proliferate and disseminate extensively. Vitamin D modulates a wide range of innate immune cells, including macrophages, dendritic cells, and natural killer (NK) cells through multiple mechanisms. This modulation not only markedly enhances the innate immune response but also alters the TME, thereby creating more conducive conditions for the application of ICIs in lung cancer treatment and significantly improving their efficacy.

#### 5.1.1. Myeloid-Derived Suppressor Cells (MDSCs)

Tumor-derived factors (TDFs) impede the normal development and differentiation of myeloid cells into mature cells, resulting in the buildup of MDSCs. These MDSCs have dual functions: facilitating tumor proliferation and assisting in tumor evasion of host immune surveillance. The principal immunosuppressive molecules implicated in these processes are arginase-1 (ARG-1), nitric oxide synthase 2 (NOS2) and prostaglandin E2 (PGE2). These two enzymes act by catabolizing L-arginine, an essential amino acid for T-cell activation, which is converted to ornithine by ARG-1, and catalyzing the production of nitric oxide, which has potent immunosuppressive properties, by NOS2. This metabolic activity ultimately leads to an effective blockade of T-cell activation. Vitamin D promotes MDSC maturation into myeloid lineage cells [53]. VDR levels within MDSCs are elevated by vitamin D, and the vitamin D signaling pathway is activated; we have found that this leads to a decrease in the enzymatic activity of ARG-1 and NOS2. Consequentially, there is a decrease in the generation of NO and L-arginine metabolism within the TME. The inhibition of MDSCs can augment anti-tumor immunity and enhance the efficacy of immunotherapy [13,54]. PGE2 is derived from arachidonic acid through the catalysis of cyclooxygenase-2 (COX-2). During the progression of lung cancer, the overexpression of COX-2 and the downregulation of 15-prostaglandin dehydrogenase (15-PGDH) result in the pathological accumulation of PGE2 in the TME. This accumulation initiates a cascade of immunosuppressive responses, impairing the standard anti-tumor immune mechanisms and facilitating tumor development and metastasis [55]. Research indicates that vitamin D regulates the expression of 15-PGDH [56]. The upregulation of 15-PGDH significantly promotes the metabolic catabolism of PGE2, thereby diminishing its accumulation in the TME. This subsequently mitigates the immunosuppressive condition and improves the effectiveness of ICIs.

#### 5.1.2. Tumor-Associated Macrophages (TAMs)

Macrophages infiltrating the TME, referred to as TAMs, form a heterogeneous immune cell population. In response to various cytokine stimuli, macrophages can polarize into two phenotypes: M1 macrophages and M2 macrophages, each displaying unique characteristics and functions. M1 macrophages possess potent anti-tumor properties, whereas M2 macrophages promote tumor proliferation and migration. Within the TME, TAMs predominantly exhibit M2-like polarization, endowing them with critical roles such as suppressing the activity of CD8^+^ T cells to facilitate immune escape [57,58,59]. PGE2 can promote the M2 polarization of macrophages. As mentioned above, vitamin D can reduce the level of PGE2, thereby decreasing the polarization of M2 TAMs [60]. Vitamin D and its derivatives can induce the production of regulatory T cells (Tregs) via the VDR and other mechanisms. Tregs can suppress the generation of TAMs, thereby inhibiting the tumor-promoting effects mediated by TAMs [61]. It was found in ovarian cancer research that vitamin D can reverse the polarization of M2 macrophages and repolarize them into M1 macrophages with anti-tumor properties. This shift in polarization effectively reduces the tumor-promoting signals in the TME, thereby exerting an inhibitory effect on tumor progression and enhancing the efficacy of immunotherapy [62]. We suppose that vitamin D may modulate the immune response within the TME by affecting the polarization state of TAMs, consequently achieving a synergistic effect in lung cancer immunotherapy. Nevertheless, additional experimental studies are needed to validate this hypothesis.

#### 5.1.3. Dendritic Cells (DCs)

Vitamin D regulates cytokine release in DCs, essential antigen-presenting cells pivotal for the initiation and modulation of immune responses. Vitamin D alters the cytokine profile in DCs by suppressing pro-inflammatory cytokines like IL-12 and IL-23, while simultaneously boosting the expression of the anti-inflammatory cytokine IL-10. This alteration in cytokine release enables the conversion of DCs from a pro-inflammatory to an anti-inflammatory phenotype, thereby diminishing inflammatory responses within the TME [52]. Comprehensive research has demonstrated that this inflammatory condition fosters an environment favorable for tumor proliferation and metastasis [63]. Thus, vitamin D enhances the effectiveness of ICIs by improving the inflammatory state of the TME.

#### 5.1.4. Natural Killer Cells (NK Cells)

NK cells are vital for initiating early anti-tumor immune responses. Vitamin D enhances the antibody-dependent cellular cytotoxicity (ADCC) function of NK cells through the upregulation of specific interferon α (IFN-α) subtypes and interferon κ expression levels. Research indicates that vitamin D consumption markedly improves the cytotoxic function of NK cells, resulting in more efficient eradication of tumor cells [64]. Through this mechanism, vitamin D enhances the activity of NK cells, which in turn boosts the efficacy of ICI therapy, presenting new strategies and possibilities for lung cancer treatment.

#### 5.1.5. Modulation of the Microbiome

A collaborative study conducted by the Francis Crick Institute in the United Kingdom and other institutions uncovered that vitamin D can augment the efficacy of ICIs by means of modulation of the intestinal microbiome, particularly by promoting the growth of Bacteroides fragilis. The researchers observed that mice with higher levels of vitamin D exhibited a more favorable response to cancer immunotherapy [65]. This finding underscores the potential for vitamin D as a significant factor influencing tumor-immune status and the efficacy of immunological therapy. Intestinal microbial communities are essential for immune maturation and homeostasis. Vitamin D’s modulation of the intestinal microbiome may reconfigure the gut microecological environment, thereby impacting the differentiation, activation, and functionality of immune cells. This interaction could synergize with ICIs to improve the anti-tumor immune response. Further investigation is warranted to confirm whether this mechanism potentially heightens the potency of ICIs in lung carcinoma patients.

### 5.2. Adaptive Immunity System

The adaptive immune system is predominantly composed of T cells and B cells. T cells serve as a central component in tumor immunity, particularly cytotoxic T lymphocytes (CTLs), which possess the ability to directly identify and eliminate cancer cells expressing tumor antigens. ICIs represent a class of therapeutic agents designed to augment the immune response by inhibiting immune checkpoint molecules. The efficacy of ICIs is largely contingent upon the status of immune cells within the TME. Vitamin D contributes to alleviating immunosuppression in the TME by promoting T cell differentiation and activation, as well as by modulating cytokine secretion from Tregs. This action enhances the effectiveness of ICIs. Consequently, vitamin D and ICIs may exhibit synergistic effects, potentially improving treatment outcomes and prognoses for lung cancer patients.

#### 5.2.1. The Activation of CD8^+^ T Lymphocytes

Research conducted by Professor Carsten Geisler and his team demonstrated that in the naive T cell state, the expression level of the VDR is relatively low. Once the TCR is activated, the p38/mitogen-activated protein kinase (MAPK) signaling cascade is triggered, leading to the induction of VDR expression. Following this, vitamin D binds to VDR, eliciting the upregulation of phospholipase C-γ1 (PLC-γ1) expression via VDR. Following the upregulation of PLC-γ1 expression, its capacity to hydrolyze phosphatidylinositol-4,5-bisphosphate (PIP_2_) is significantly enhanced, thereby leading to more efficient production of inositol 1,4,5-trisphosphate (IP_3_). IP_3_ can further activate the Ca^2+^-releasing channel in the endoplasmic reticulum (ER), inducing the release of Ca^2+^ from this organelle [66]. Preclinical investigations have established that the binding of vitamin D to the VDR activates Ca^2+^ channels in T cells, thereby promoting Ca^2+^ influx. This process is highly dependent on VDR function [12]. As an important intracellular messenger, the elevated Ca^2+^ concentration enhances TCR signaling and T cell activation. The promotion of calcium release and influx from the endoplasmic reticulum increases Ca^2+^ concentration, which activates T cells and strengthens the anti-tumor immune response, thereby augmenting the efficacy of ICIs.

#### 5.2.2. Mitigate the Depletion of CD8^+^ T Cells Within the TME

In the TME, CD8^+^ T cells progressively transition into an exhausted state due to continuous antigenic stimulation. This process is marked by the enhanced expression of co-inhibitory receptors, including PD-1, TIGIT, and TIM-3. The upregulation of these receptors inhibits cellular activity, leading to a gradual decline in their cytotoxic and proliferative functions. Ultimately, these cells are transformed into exhausted CD8^+^ T effector (Tex) cells [67]. Immune checkpoint blockers (ICBs) can partially inhibit the co-inhibitory signaling pathway, thereby rescuing certain PD-1 expressing cells from a non-responsive or exhausted state. Research findings have substantiated that, within the context of anti-PD-L1/PD-1 therapy, only recently exhausted T cells featuring relatively low expression of PD-1 show the potential for functional restoration. In contrast, over-exhausted effector T cells show higher PD-1 expression alongside other activation markers (e.g., TIM-3, TIGIT) and demonstrate limited potential for restoring activity [68,69]. In patients with lung carcinoma, plasma vitamin D levels exhibit an inverse correlation with the expression of co-inhibitory immune checkpoints and a positive correlation with the expression of the co-stimulatory molecule CD28. This relationship is primarily mediated through gene regulation and epigenetic modifications, ultimately resulting in decreased PD-1 expression and increased CD28 expression [12]. Vitamin D can efficiently restore T cell function by inhibiting PD-1 expression, which in turn enhances the efficacy of ICIs in anti-tumor responses.

#### 5.2.3. CD4^+^ T Cell

The interaction between vitamin D and the VDR can induce the expansion of Tregs, upregulate IL-10, and downregulate pro-inflammatory cytokines such as IL-17 and interferon-γ (IFN-γ). By modulating the balance of Treg/Th17 cell populations, vitamin D can reprogram the immunosuppressive characteristics of the TME, thereby creating a milieu less favorable for tumor cell proliferation and enhancing the effectiveness of immunotherapy [70]. Studies on lung adenocarcinoma have shown that the expression levels of the VDR in tumor cells are markedly elevated compared to those in adjacent normal tissues. Moreover, increased VDR expression is positively correlated with immune components within TME [71]. Therefore, the interplay between vitamin D and VDR may represent a promising approach to reshape the immune response network within the TME. This phenomenon can be elucidated by the heightened expression of chemokine receptors CCR4, CXCR3, and CCR8 on activated Tregs, which facilitates the directed migration of cytotoxic lymphocytes and antigen-presenting cells into the TME. This augments immune surveillance and the cytotoxic activity against tumor cells, thereby contributing positively to tumor immunotherapy [72].

## 6. Discussion

Within the domain of lung cancer immunotherapy, vitamin D has progressively drawn attention and demonstrated its potential value, paving the way for a novel research direction aimed at optimizing the performance of ICIs. Unlike traditional anti-tumor agents that directly induce cytotoxic effects on tumor cells, vitamin D precisely modulates the TME via multiple regulatory pathways, reshaping an immunologically “cold” TME into a responsive milieu and thereby playing an essential supportive role in the immunotherapy framework for lung cancer. This novel mechanism of action offers renewed hope to lung cancer patients who have developed resistance to immunotherapy following prolonged use. It enables the restoration of immune sensitivity to tumor cells, thereby allowing patients to potentially benefit from immunotherapy once more.

Despite the promising potential of vitamin D in lung cancer immunotherapy, numerous challenges remain to be addressed. The synergistic effects of vitamin D in immunotherapy require further clinical trials. Additionally, further research is essential to identify the most suitable dosage, administration frequency, and optimal timing for vitamin D supplementation in lung cancer immunotherapy. Potential complications from vitamin D overdose, such as hypercalcemia, also require careful consideration. Given the significant differences in tumor cell biology, immune microenvironment composition, genetic background, lifestyle, and comorbidities among different subtypes of lung cancer and individual patients, personalized approaches are essential. These factors may determine the mechanisms and overall efficacy of vitamin D. Currently, our understanding of these intricate influencing factors and underlying mechanisms remains limited.

Future studies ought to concentrate on clarifying the mechanisms of action of vitamin D in the complex in vivo environment. Furthermore, examining the synergistic effects of vitamin D in combination with ICIs at molecular, cellular, and systemic levels is critically important. This comprehensive approach will provide a robust and reliable theoretical foundation for the development of clinical treatment protocols. Such efforts will not only personalize the application of vitamin D in lung cancer immunotherapy but also enhance practical therapeutic benefits for patients, thereby maximizing the benefits of immunotherapy while minimizing risks and offering hope for improved survival rates and quality of life for lung cancer patients.

## Figures and Tables

**Table 1:** Lung cancer research related to vitamin D.

Participants	Country	Design	Follow-up	Outcomes
8 studies [41]	4 countries	Meta-analysis	10.8 months–20 years	OS
17 studies [43]	More than 5 countries	Meta-analysis	NA	OS
155 patients [44]	Japan	A randomized, double-blind trial	1 year	OS
77 patients [48]	China	A prospective study	More than 1 year	OS, PFS
45 patients [49]	Italy	A clinical trial	60 days	The level of 25(OH)D and nivolumab

OS: Overall Survival; PFS: Progression-Free Survival; NA: Not Available.

**Table 2:** Clinical studies related to immunotherapy and vitamin D.

References	Design	Participants	Cohorts	Main Findings	Conclusions
[15]	A prospective controlled study	200 patients with advanced melanoma	**∙** Group A*: Reduced VitD levels **∙** Group B*: Normal VitD levels	**∙** ORR 36.2%; mPFS 5.6 months **∙** ORR 56%; mPFS 11.25 months	Maintaining normal VitD levels may improve ICI efficacy
[16]	A prospective controlled study	164 patients with advanced cancer	**∙** Cohort 1: 101 patients (anti-PD-1 + early VitD repletion) **∙** Cohort 2: 63 patients (anti-PD-1 + delayed VitD repletion) **∙** Control cohort: 238 patients (without systematic VitD repletion)	**∙** mOS 15.9 months; mTTF 5.2 months; ORR 33.3% **∙** mOS not achieved ; mTTF 23.2 months; ORR 33.3% **∙** mOS 7.1 months; mTTF 3.1 months; ORR 25%	Early VitD repletion may improve outcomes in advanced cancer patients on ICIs
[48]	A prospective controlled study	77 patients with advanced lung cancers	Baseline VitD level **∙** Sufficiency (<10 ng/mL) **∙** Insufficiency (10–20 ng/mL) **∙** Deficiency (≥20 ng/mL)	**∙** mPFS 606 days VS 326 days VS 308 days **∙** PR patients’ VitD baseline level higher than non-PR patients **∙** irAE 37%	**∙** Baseline VitD levels may correlate with ICI efficacy and prognosis **∙** Supplementing VitD might enhance ICI efficacy and reduce moderate-to-severe irAEs
[49]	An observational study	45 patients with advanced lung cancers	The level of Nivolumab and VitD **∙** Day 0 **∙** Day 15 **∙** Day 30 **∙** Day 45	**∙** 25(OH)D_3_ 12.8 ng/mL (D0) 13.6 ng/mL (D15) 11.8 ng/mL (D30) 12.9 ng /mL(D45) **∙** Nivolumab 12.5 μg/mL (D15) 22.3 μg/mL (D30) 27.1 μg/mL (D45)	The concentration of nivolumab is correlated with the VitD level
[50]	A retrospective study	213 melanoma patients who received ICIs and developed irAE	**∙** The discovery cohort (213 patients) **∙** The confirmatory cohort (169 patients)	Compared with patients not taking vitamin D **∙** ICI-related colitis decreased by 65% **∙** ICI-related colitis decreased by 54%	Patients using VitD had significantly decreased likelihood of developing ICI-related colitis

Group A*: reduced vitamin D levels include baseline vitamin D < 30 ng/mL and remained < 30 ng/mL throughout the treatment period, regardless of supplementation; Group B*: normal vitamin D levels include baseline vitamin D ≥ 30 ng/mL or <30 ng/mL but reached ≥30 ng/mL after vitamin D supplementation. Both groups of patients received anti-PD-1 treatment; Day 0, Day 15, Day 30, and Day 45: measured the levels of Nivolumab and Vitamin D prior to initiating treatment and at 15, 30, and 45 days into therapy; ORR: objective response rate; mTTF: median time to failure; mOS: median overall survival; mPFS: median progression-free survival; PR: partial response; irAE: immune-related adverse events; VitD: vitamin D.

## Data Availability

The data related to this study are available in this article and Supplementary Materials [19,73,74,75,76,77,78,79,80,81,82,83,84,85,86,87,88,89,90,91,92,93,94,95,96,97,98,99,100,101,102,103,104,105,106,107].

## Supplementary Materials

The following supporting information can be downloaded at: https://www.mdpi.com/article/10.3390/ijms26104511/s1.
