# Supplementary material for: Vitamin D and Immune Checkpoint Inhibitors in Lung Cancer: A Synergistic Approach to Enhancing Treatment Efficacy
Source: Int J Mol Sci. 2025 May 9;26(10):4511. doi: 10.3390/ijms26104511 (PMC12111780; doi:10.3390/ijms26104511)
Supplement: Supplementary file 1 [file ijms-26-04511-s001.zip › ijms-3559134-supplementary.pdf]

Supplementary Table 1. Research on immune checkpoint inhibitors in lung cancer

References	ICI	Target	Structure Type	Approved Indications	Approved Regions
[1–3]	Nivolumab	PD-1	Fully human IgG4 monoclonal	・Neoadjuvant/adjuvant Nivolumab for resectable NSCLC<br>・Advanced NSCLC	Global (US, EU, China)
[4–6]	Pembrolizumab	PD-1	Humanized IgG4 monoclonal	・Neoadjuvant/adjuvant Nivolumab for resectable NSCLC<br>・Advanced NSCLC	Global (US, EU, China)
[7–9]	Sintilimab	PD-1	Fully human IgG4 monoclonal	・Advanced NSCLC<br>・Advanced non-squamous NSCLC patients with positive EGFR mutations who failed EGFR-TKI therapy	China
[10–12]	Camrelizumab	PD-1	Humanized IgG4 monoclonal	・Advanced NSCLC	China
[13–15]	Tislelizumab	PD-1	Humanized IgG4 monoclonal	・Neoadjuvant/adjuvant Nivolumab for resectable NSCLC<br>・Advanced NSCLC	Global (US, EU, China)
[16–18]	Toripalimab	PD-1	Humanized IgG4 monoclonal	・Neoadjuvant/adjuvant Nivolumab for resectable NSCLC<br>・Advanced NSCLC<br>・ES-SCLC	China, US
[19,20]	Serplulimab	PD-1	Humanized IgG4 monoclonal	・Advanced squamous NSCLC<br>・ES-SCLC	China
[21–23]	Atezolizumab	PD-L1	Humanized IgG1 monoclonal	・Advanced NSCLC<br>・ES-SCLC	Global (US, EU, China)
[24–27]	Durvalumab	PD-L1	Humanized IgG1 monoclonal	・Neoadjuvant/adjuvant Nivolumab for resectable NSCLC<br>・Advanced NSCLC<br>・ES-SCLC<br>・LS-SCLC	Global (US, China)
[28]	Envafolimab	PD-L1	Humanized IgG1 monoclonal	・Advanced NSCLC	China
[29]	Sugemalimab	PD-L1	Fully human IgG4 monoclonal	・Advanced NSCLC	China
[30]	Adebrelimab	PD-L1	Humanized IgG4 monoclonal	・ES-SCLC	China
[31]	Benmelstobart	PD-L1	Humanized IgG1 monoclonal	・ES-SCLC	China

[32]	Ipilimumab	CTLA-4	Fully Humanized IgG1 monoclonal	· Advanced NSCLC	Global (US, EU, China)
[33]	Tremelimumab	CTLA-4	Fully Humanized IgG2 monoclonal	· Advanced NSCLC	Global (US, EU)
[34]	Cadonilimab	PD-1/CTLA-4	Humanized IgG1 monoclonal	· Clinical trials	___
[35]	Envafolimab	PD-1/VEGF	Humanized IgG1 monoclonal	· Clinical trials	___
[36]	Iparomlimab	PD-1/CTLA-4	Humanized IgG4 monoclonal	· Clinical trials	___

NSCLC: non-small cell lung cancer; ES-SCLC：extensive-stage small-cell lung cancer: LS-SCLC：limited-stage small cell lung cancer.

References

1. Cascone, T.; Awad, M.M.; Spicer, J.D.; He, J.; Lu, S.; Sepesi, B.; Tanaka, F.; Taube, J.M.; Cornelissen, R.; Havel, L.; *et al*. Perioperative Nivolumab in Resectable Lung Cancer. *N. Engl. J. Med.* **2024**, *390*, 1756–1769, doi:10.1056/NEJMoa2311926.
2. Borghaei, H.; Gettinger, S.; Vokes, E.E.; Chow, L.Q.M.; Burgio, M.A.; de Castro Carpeno, J.; Pluzanski, A.; Arrieta, O.; Frontera, O.A.; Chiari, R.; *et al*. Five-Year Outcomes from the Randomized, Phase III Trials CheckMate 017 and 057: Nivolumab versus Docetaxel in Previously Treated Non-Small-Cell Lung Cancer. *J. Clin. Oncol.* **2021**, *39*, 723–733, doi:10.1200/JCO.20.01605.
3. Forde, P.M.; Spicer, J.; Lu, S.; Provencio, M.; Mitsudomi, T.; Awad, M.M.; Felip, E.; Broderick, S.R.; Brahmer, J.R.; Swanson, S.J.; *et al*. Neoadjuvant Nivolumab plus Chemotherapy in Resectable Lung Cancer. *N. Engl. J. Med.* **2022**, *386*, 1973–1985, doi:10.1056/NEJMoa2202170.
4. Spicer, J.D.; Garassino, M.C.; Wakelee, H.; Liberman, M.; Kato, T.; Tsuboi, M.; Lee, S.-H.; Chen, K.-N.; Dooms, C.; Majem, M.; *et al*. Neoadjuvant Pembrolizumab plus Chemotherapy Followed by Adjuvant Pembrolizumab Compared with Neoadjuvant Chemotherapy Alone in Patients with Early-Stage Non-Small-Cell Lung Cancer (KEYNOTE-671): A Randomised, Double-Blind, Placebo-Controlled, Phase 3 Trial. *Lancet.* **2024**, *404*, 1240–1252 ，doi:10.1016/S0140-6736(24)01756-2.
5. Herbst, R.S.; Garon, E.B.; Kim, D.-W.; Cho, B.C.; Gervais, R.; Perez-Gracia, J.L.; Han, J.-Y.; Majem, M.; Forster, M.D.; Monnet, I.; *et al*. Five Year Survival Update from KEYNOTE-010: Pembrolizumab versus Docetaxel for Previously Treated, Programmed Death-Ligand 1–Positive Advanced NSCLC. *J. Thorac. Oncol.* **2021**, *16*, 1718–1732，doi:10.1016/j.jtho.2021.05.001.
6. Novello, S.; Kowalski, D.M.; Luft, A.; Gümüş, M.; Vicente, D.; Mazières, J.; Rodríguez-Cid, J.; Tafreshi, A.; Cheng, Y.; Lee, K.H.; *et al*. Pembrolizumab plus Chemotherapy in Squamous Non-Small-Cell Lung Cancer: 5-Year Update of the Phase III KEYNOTE-407 Study. *J. Clin. Oncol.* **2023**, *41*, 1999–2006, doi:10.1200/JCO.22.01990.

7. L,S.; W,L.; J,H.; C,Y. W,Q.M.;F,J.; W,Z.P.; H,Y.P.; *et al*. Sintilimab plus Chemotherapy for Patients with EGFR-Mutated Non-Squamous Non-Small-Cell Lung Cancer with Disease Progression after EGFR Tyrosine-Kinase Inhibitor Therapy (ORIENT-31): Second Interim Analysis from a Double-Blind, Randomised, Placebo-Controlled, Phase 3 Trial. *Lancet. Respir. Med*. 2023,11,624-636, doi: 10.1016/S2213-2600(23)00135-2.

8. Xie, J.; Wu, X.; Wu, J.; Huang, F.; Xu, L. Meta-Analysis of the Efficacy and Safety of Sintilimab for Treating Advanced Non-Small Cell Lung Cancer. *Oncol. Lett.* **2022**, *24*, 425, doi:10.3892/ol.2022.13545.

9. Zhang, M.; Zhang, G.; Niu, Y.; Zhang, G.; Ji, Y.; Yan, X.; Zhang, X.; Wang, Q.; Jing, X.; Wang, J.; *et al*. Sintilimab with Two Cycles of Chemotherapy for the Treatment of Advanced Squamous Non-Small Cell Lung Cancer: A Phase 2 Clinical Trial. *Nat Commun.* **2024**, *15*, 1512, doi:10.1038/s41467-024-45769-z.

10. Ren, S.; Chen, J.; Xu, X.; Jiang, T.; Cheng, Y.; Chen, G.; Pan, Y.; Fang, Y.; Wang, Q.; Huang, Y.; *et al*. Camrelizumab plus Carboplatin and Paclitaxel as First-Line Treatment for Advanced Squamous NSCLC (CameL-Sq): A Phase 3 Trial. *J. Thorac. Oncol.* **2022**, *17*, 544–557, doi:10.1016/j.jtho.2021.11.018.

11. Zhou, C.; Chen, G.; Huang, Y.; Zhou, J.; Lin, L.; Feng, J.; Wang, Z.; Shu, Y.; Shi, J.; Hu, Y.; *et al*. Camrelizumab plus Carboplatin and Pemetrexed as First-Line Therapy for Advanced Non-Squamous Non-Small-Cell Lung Cancer: 5-Year Outcomes of the CameL Randomized Phase 3 Study. *J. ImmunoTher. Cancer.* **2024**, *12*, e009240, doi:10.1136/jitc-2024-009240.

12. Xu, C.-R.; Chen, Q.; Zhou, C.; Wu, L.; Li, W.; Zhang, H.; Li, Y.; Xu, F.; Xiong, J.; Wang, Q.; *et al*. Effectiveness and Safety of Camrelizumab in Inoperable or Advanced Non-Small Cell Lung Cancer Patients: A Multicenter Real-World Retrospective Observational Study (CTONG2004-ADV). *Transl. Lung Cancer Res.* **2023**, *12*, 127–140, doi:10.21037/tlcr-22-852.

13. Yue, D.; Wang, W.; Liu, H.; Chen, Q.; Chen, C.; Liu, L.; Zhang, P.; Zhao, G.; Yang, F.; Han, G.; *et al*. Perioperative Tislelizumab plus Neoadjuvant Chemotherapy for Patients with Resectable Non-Small-Cell Lung Cancer (RATIONALE-315): An Interim Analysis of a Randomised Clinical Trial. *Lancet Respir. Med.* **2025**, *13*, 119–129, doi:10.1016/S2213-2600(24)00269-8.

14. Daei Sorkhabi, A.; ZareDini, M.; Fazlollahi, A.; Sarkesh, A.; Naseri, A.; Mousavi, S.E.; Nejadghaderi, S.A.; Sullman, M.J.M.; Kolahi, A.-A.; Safiri, S. The Safety and Efficacy of Tislelizumab, Alone or in Combination with Chemotherapy, for the Treatment of Non-Small Cell Lung Cancer: A Systematic Review of Clinical Trials. *BMC Pulm. Med.* **2023**, *23*, 495, doi:10.1186/s12890-023-02755-3.

15. Ul Bassar, W.; Ogedegbe, O.J.; Qammar, A.; Sumia, F.; Ul Islam, M.; Chaudhari, S.S.; Ntukidem, O.L.; Khan, A. Efficacy of Tislelizumab in Lung Cancer Treatment: A Systematic Review and Meta-Analysis of Randomized Controlled Trials. *Cureus. 2025, 17*, e80609, doi:10.7759/cureus.80609.

16. Liu, Y.; Yang, L.; Duan, Z.; Cheng, Q.; Liu, M.; Zhang, H.; Zhao, H. Efficacy and Safety of Toliparibumab for the Treatment of Non-Small Cell Lung Cancer: A Systematic Review and Meta-Analysis. *Front. Oncol.* **2024**, *14*, 1444312, doi:10.3389/fonc.2024.1444312.

17. Zhong, J.; Fei, K.; Wu, L.; Li, B.; Wang, Z.; Cheng, Y.; Li, X.; Wang, X.; Han, L.; Wu, X.; *et al*. Toripalimab plus Chemotherapy for First Line Treatment of Advanced Non-Small Cell Lung

Cancer (CHOICE-01): Final OS and Biomarker Exploration of a Randomized, Double-Blind, Phase 3 Trial. *Signal Transduction Targeted Ther.* **2024**, *9*, 369, doi:10.1038/s41392-024-02087-6.
18. Cheng, Y.; Zhang, W.; Wu, L.; Zhou, C.; Wang, D.; Xia, B.; Bi, M.; Fu, X.; Li, C.; Lv, D.; *et al*. Toripalimab plus Chemotherapy as a First-Line Therapy for Extensive-Stage Small Cell Lung Cancer: The Phase 3 EXTENTORCH Randomized Clinical Trial. *JAMA Oncol.* **2025**, *11*, 16, doi:10.1001/jamaoncol.2024.5019.
19. Cheng, Y.; Han, L.; Wu, L.; Chen, J.; Sun, H.; Wen, G.; Ji, Y.; Dvorkin, M.; Shi, J.; Pan, Z.; *et al*. Effect of First-Line Serplulimab vs Placebo Added to Chemotherapy on Survival in Patients with Extensive-Stage Small Cell Lung Cancer: The ASTRUM-005 Randomized Clinical Trial. *JAMA.* 2022, *328*, 1223, doi:10.1001/jama.2022.16464.
20. Zhou, C.; Hu, Y.; Arkania, E.; Kilickap, S.; Ying, K.; Xu, F.; Wu, L.; Wang, X.; Viguro, M.; Makharadze, T.; *et al*. A Global Phase 3 Study of Serplulimab plus Chemotherapy as First-Line Treatment for Advanced Squamous Non-Small-Cell Lung Cancer (ASTRUM-004). *Cancer Cell.* 2024, *42*, 198-208.e3, doi:10.1016/j.ccell.2023.12.004.
21. West, H.; McCleod, M.; Hussein, M.; Morabito, A.; Rittmeyer, A.; Conter, H.J.; Kopp, H.-G.; Daniel, D.; McCune, S.; Mekhail, T.; *et al*. Atezolizumab in Combination with Carboplatin plus Nab-Paclitaxel Chemotherapy Compared with Chemotherapy Alone as First-Line Treatment for Metastatic Non-Squamous Non-Small-Cell Lung Cancer (IMpower130): A Multicentre, Randomised, Open-Label, Phase 3 Trial. *Lancet Oncol.* 2019, *20*, 924–937, doi:10.1016/S1470-2045(19)30167-6.
22.Fehrenbacher, L.; Spira, A.; Ballinger, M.; Kowanetz, M.; Vansteenkiste, J.; Mazieres, J.; Park, K.; Smith, D.; Artal-Cortes, A.; Lewanski, C.; et al. Atezolizumab versus Docetaxel for Patients with Previously Treated Non-Small-Cell Lung Cancer (POPLAR): A Multicentre, Open-Label, Phase 2 Randomised Controlled Trial. *Lancet.* 2016, *387*, 1837–1846, doi:10.1016/S0140-6736(16)00587-0.
23. Liu, S.V.; Reck, M.; Mansfield, A.S.; Mok, T.; Scherpereel, A.; Reinmuth, N.; Garassino, M.C.; De Castro Carpeno, J.; Califano, R.; Nishio, M.; *et al*. Updated Overall Survival and PD-L1 Subgroup Analysis of Patients with Extensive-Stage Small-Cell Lung Cancer Treated with Atezolizumab, Carboplatin, and Etoposide (IMpower133). *J. Clin. Oncol.* 2021, *39*, 619–630, doi:10.1200/JCO.20.01055.
24. Barlesi, F.; Cho, B.C.; Goldberg, S.B.; Yoh, K.; Zimmer Gelatti, A.C.; Mann, H.; Gopinathan, A.; Bielecka, Z.F.; Newton, M.; Aggarwal, C. PACIFIC-9: Phase III Trial of Durvalumab + Oleclumab or Monalizumab in Unresectable Stage III Non-Small-Cell Lung Cancer. *Future Oncol.* 2024, *20*, 2137–2147, doi:10.1080/14796694.2024.2354160.
25. Cheng, Y.; Spigel, D.R.; Cho, B.C.; Laktionov, K.K.; Fang, J.; Chen, Y.; Zenke, Y.; Lee, K.H.; Wang, Q.; Navarro, A.; *et al*. Durvalumab after Chemoradiotherapy in Limited-Stage Small-Cell Lung Cancer. *N. Engl. J. Med.* 2024, *391*, 1313–1327, doi:10.1056/NEJMoa2404873.
26. Heymach, J.V.; Harpole, D.; Mitsudomi, T.; Taube, J.M.; Galffy, G.; Hochmair, M.; Winder, T.; Zukov, R.; Garbaos, G.; Gao, S.; *et al*. Perioperative Durvalumab for Resectable Non–Small-Cell Lung Cancer. *N. Engl. J. Med.* 2023, *389*, 1672–1684, doi:10.1056/NEJMoa2304875.
27. Paz-Ares, L.; Dvorkin, M.; Chen, Y.; Reinmuth, N.; Hotta, K.; Trukhin, D.; Statsenko, G.; Hochmair, M.J.; Özgüroğlu, M.; Ji, J.H.; *et al*. Durvalumab plus Platinum–Etoposide versus Platinum–Etoposide in First-Line Treatment of Extensive-Stage Small-Cell Lung Cancer

(CASPIAN): A Randomised, Controlled, Open-Label, Phase 3 Trial. *Lancet.* 2019, *394*, 1929–1939, doi:10.1016/S0140-6736(19)32222-6.
28. Papadopoulos, K.P.; Harb, W.; Peer, C.J.; Hua, Q.; Xu, S.; Lu, H.; Lu, N.; He, Y.; Xu, T.; Dong, R.; *et al*. First-in-human Phase I Study of Envafolimab, a Novel Subcutaneous Single-domain anti-PD-L1 Antibody, in Patients with Advanced Solid Tumors. *Oncologist.* 2021, *26*, e1514–e1525, doi:10.1002/onco.13817.
29. Zhou, C.; Wang, Z.; Sun, M.; Cao, L.; Ma, Z.; Wu, R.; Yu, Y.; Yao, W.; Sun, S.; Chen, J.; *et al*. Interim Survival Analysis of the Randomized Phase III GEMSTONE-302 Trial: Sugemalimab or Placebo plus Chemotherapy as First-Line Treatment for Metastatic NSCLC. *Nat. Cancer.* 2023, *4*, 860–871， doi:10.1038/s43018-023-00578-z.
30. Gan, Y.; Shi, F.; Zhu, H.; Han, S.; Li, D. Adebrelimab plus Chemotherapy vs. Chemotherapy for Treatment of Extensive-Stage Small-Cell Lung Cancer from the US and Chinese Healthcare Sector Perspectives: A Cost-Effectiveness Analysis to Inform Drug Pricing. *Front. Pharmacol.* 2023, *14*, 1241130, doi:10.3389/fphar.2023.1241130.
31. Cheng, Y.; Chen, J.; Zhang, W.; Xie, C.; Hu, Q.; Zhou, N.; Huang, C.; Wei, S.; Sun, H.; Li, X.; *et al*. Benmelstobart, Anlotinib and Chemotherapy in Extensive-Stage Small-Cell Lung Cancer: A Randomized Phase 3 Trial. *Nat. Med.* 2024, *30*, 2967–2976, doi:10.1038/s41591-024-03132-1.
32. Paz-Ares, L.; Ciuleanu, T.E.; Cobo, M.; Schenker, M.; Zurawski, B.; Menezes, J.; Richardet, E.; Bennouna, J.; Felip, E.; Juan-Vidal, O.; *et al*. First-Line Nivolumab plus Ipilimumab Combined with Two Cycles of Chemotherapy in Patients with Non-Small-Cell Lung Cancer (CheckMate 9LA): An International, Randomised, Open-Label, Phase 3 Trial. *Lancet Oncol.* 2021, *22*, 198–211, doi:10.1016/S1470-2045(20)30641-0.
33. Johnson, M.L.; Cho, B.C.; Luft, A.; Alatorre-Alexander, J.; Geater, S.L.; Laktionov, K.; Kim, S.-W.; Ursol, G.; Hussein, M.; Lim, F.L.; *et al*. Durvalumab with or without Tremelimumab in Combination with Chemotherapy as First-Line Therapy for Metastatic Non–Small-Cell Lung Cancer: The Phase III POSEIDON Study. *J. Clin. Oncol.* 2023, *41*, 1213–1227, doi:10.1200/JCO.22.00975.
34. Gao, X.; Xu, N.; Li, Z.; Shen, L.; Ji, K.; Zheng, Z.; Liu, D.; Lou, H.; Bai, L.; Liu, T.; *et al*. Safety and Antitumour Activity of Cadonilimab, an Anti-PD-1/CTLA-4 Bispecific Antibody, for Patients with Advanced Solid Tumours (COMPASSION-03): A Multicentre, Open-Label, Phase 1b/2 Trial. *Lancet Oncol.* 2023, *24*, 1134–1146, doi:10.1016/S1470-2045(23)00411-4.
35. Ma, L.X.; Zhang, Y.; Fang, Y.; Hao, C.; Fan, Q.; Jiang, D.; Lu, L.; Su, F.; Yang, C.; Liu, Z.; *et al*. Anti-PD-L1 Envafolimab Combined with Anti-VEGF Suvemcitug in Pretreated Solid Tumors and Hepatocellular Carcinoma: An Open-Label Phase II Study with Safety Run-in Stage. *Invest. New Drugs.* 2025, doi:10.1007/s10637-025-01506-x.
36. Huang, Y.; Yang, Y.; Zhao, Y.; Zhao, H.; Zhou, N.; Zhang, Y.; Chen, L.; Zhou, T.; Chen, G.; Wu, T.; *et al*. QL1706 (Anti-PD-1 IgG4/CTLA-4 Antibody) plus Chemotherapy with or without Bevacizumab in Advanced Non-Small Cell Lung Cancer: A Multi-Cohort, Phase II Study. *Signal Transduction Targeted Ther.* 2024, *9*, 23, doi:10.1038/s41392-023-01731-x.
